# Pd-Catalyzed Cyclocarbonylation of Allylic Alcohol under Benign Conditions with Ionic Liquid as Stabilizer

**DOI:** 10.3390/ma13092093

**Published:** 2020-05-01

**Authors:** Nasrin Nemati, Reza Eslamloueyan, Amalie Modvig, Anders Riisager

**Affiliations:** 1 Department of Chemical Engineering, School of Chemical and Petroleum Engineering, Shiraz University, Shiraz 71345, Iran; nasrinn@chalmers.se; 2 Centre for Catalysis and Sustainable Chemistry, Department of Chemistry, Technical University of Denmark, DK-2800 Kgs. Lyngby, Denmark; amaliemodvig@gmail.com

**Keywords:** allylic alcohol, ionic liquid, Pd-diphosphine catalyst, cyclocarbonylation, lactone production, catalyst stabilization and recycling

## Abstract

Homogeneous palladium-catalyzed (Pd-catalyzed) cyclocarbonylation of unsaturated allylic alcohols and alkynols in the presence of hydrogen forms lactone products with important applications in the food, perfume, and polymer industry. In this work, the cyclocarbonylation of 2-methyl-3-buten-2-ol was studied for the first time using a very active Pd-DPEphos (bis[(2-diphenylphosphino)phenyl]ether) catalyst in the presence of the ionic liquid (IL) [BMIM]Cl (1-butyl-3-methylimidazolium chloride) in dichloromethane to selectively produce 4,4-dimethyl-γ-butyrolactone. The effect of different parameters such as temperature, gas partial pressures, time of reaction, substrate and ligand concentrations were investigated and found to provide optimal conditions for lactonization (95 °C, 28 bar (CO/H_2_/N_2_: 20/5/3)), 18 h, 0.1 M substrate, and 16 mol% DPEphos), which were significantly milder than previously reported systems for cyclocarbonylation. Importantly, the study further showed that presence of the IL in the reaction mixture provided stabilization of the catalyst system and prevented formation of Pd-black, which allowed reuse of the catalytic system in consecutive reactions after intermediate extraction of the lactone product.

## 1. Introduction

Metal-catalyzed cyclocarbonylations of unsaturated allylic alcohols or alkynols with carbon monoxide are atom efficient synthetic routes for producing important chemicals like lactones and indanones [1]. In particular, the formation of five- and six-membered (γ and δ) lactones is facile and has been widely investigated under acidic or neutral conditions using palladium (Pd) complex-based catalysts in homogeneous liquid-phase reactions [2,3,4,5,6]. In contrast, three- and four-membered (α and β) lactones are mostly reactive and short-lived intermediates, which cannot be isolated easily and require specific production routes [7,8,9,10].

Several reviews are available in the literature on reactions and mechanisms of transition-metal catalyzed carbonylation and cyclocarbonylation for lactone production using Group 8–10 catalysts, including [Fe(CO)_5_]^−^, [Co_2_(CO)_8_], [Ni(cod)_2_PCy_3_] (cod: 1,5-cyclooctadiene, PCy_3_: tricyclohexyl-phosphine), [(PPh_3_)_2_N][Co(CO)_4_] ((PPh_3_)_2_N: bis(triphenylphosphoranylidene)ammonium), and the aluminum-salen complex [(salph)Al(THF)_2_][Co(CO)_4_]) (salph: *N,N’*-bis(3,5-di-tert-butylsalicylidene)-phenylenediamino, THF: tetrahydrofuran) [10,11,12,13,14,15,16]. With noble-metal catalysts, Pd has played an almost exclusive role in lactonization reactions as it generally provides higher yields of lactones compared to other catalysts [13,14]. In particular, an interesting cyclocarbonylation catalyst system based on Pd-dppb (dppb: 1,4-bis-(diphenylphosphino)butane) was introduced by Ali and Alper [1] and Brunner and Alper [17] for the formation of γ-lactones from secondary-, tertiary- as well as β,γ-substituted allylic alcohols under neutral but quite harsh reaction conditions (40–54 bar CO/H_2_, 110–190 °C, 18–48 h). They showed that the Pd-dppb system can catalyze the reaction with good activity, for example, the allylic alcohol 2-methyl-3-buten-2-ol into 4,4-dimethyl-γ-butyrolactone (5,5-dimethyl-dihydro-furan-2-one). This product has important applications in the food, perfume, and polymer industry [1]. However, in their system, the catalyst was not recoverable or prone to reuse due to formation of catalytically inactive Pd metal (i.e., Pd-black). This is highly undesired using an expensive metal inventory such a Pd, where easy catalyst recovery and reusability as well as stabilization of the reaction system is strongly preferred [18]. Furthermore, introduction of an alternative Pd-ligand catalyst system possessing higher catalytic activity would facilitate milder reaction conditions. 

During the last two decades, ionic liquids (ILs) have demonstrated to be efficient reaction media for transforming homogeneously catalyzed processes, including carbonylations, into catalytically active biphasic liquid–liquid systems with the IL providing immobilization and stabilization of metal-complex catalysts [19,20,21,22]. Other solvent advantages of ILs are high thermal and chemical stability as well as negligible low vapor pressures, which facilitate their reuse in catalyst systems with only a requirement of small amounts of replacement, thus making the processes greener [23,24,25,26,27,28,29,30]. Ye and Alper introduced the ILs [BMIM][PF_6_] and [BMIM][NTf_2_] (BMIM: 1-butyl-3-methylimidazolium, NTf_2_: bis(trifluoromethane)sulfonimide) for cyclocarbonylation of 2-allylphenols and anilines of 2-vinylphenols and 2-aminostyrenes [31]. In the reactions using these ILs, lactones and lactams were produced with still a high total pressure of CO/H_2_ (42 bar) but at lower temperatures (90–120 °C) compared to previously described Pd-dppb catalytic systems. Notably, the cheaper and hydrolysis stable IL [BMIM]Cl alone did not facilitate any cyclocarbonylation reaction which otherwise could have been preferred.

**Scheme 1:**
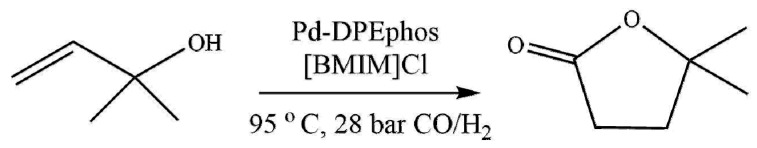
Cyclocarbonylation of 2-methyl-3-buten-2-ol to 4,4-dimethyl-γ-butyrolactone with Pd-DPEphos (DPEphos: bis[(2-diphenylphosphino)phenyl]ether) catalyst and CO/H_2_ in the presence of the ionic liquid [BMIM]Cl (1-butyl-3-methylimidazolium chloride).

In this work, a Pd-DPEphos (DPEphos: bis[(2-diphenylphosphino)phenyl]ether) catalyst system containing the IL [BMIM]Cl is for the first time reported for the selective cyclocarbonylation of 2-methyl-3-buten-2-ol into 4,4-dimethyl-γ-butyrolactone under benign reaction conditions (Scheme 1). We found that the optimal operating conditions for lactonization in this system are 95 °C, 28 bar (CO/H_2_/N_2_: 20/5/3) and 18 h, which are much milder compared to what is reported and discussed earlier in the literature [1,17,31]. Furthermore, in combination the ligand and the IL give a stabilized Pd-catalyst system, which is highly active for cyclocarbonylation reactions. Importantly, the precious Pd-catalyst is also reusable after intermediate product recovery by extraction. Employing IL in the catalytic system and providing the stability, facile catalyst recovery and its re-usability are the other main differences of this work compared to previous investigations on this reaction. 

## 2. Materials and Methods 

### 2.1. Chemicals

Palladium(II) acetate (Pd(OAc)_2_, ≥99.9%), bis[(2-diphenylphosphino)phenyl]ether (DPEphos, 98%), 2-methyl-3-buten-2-ol (98%), and 1-butyl-3-methylimidazolium chloride ([BMIM]Cl, ≥98%) were obtained from Sigma-Aldrich (Søborg, Denmark) and diethyl ether (DEE, ≥99% GPR RECTAPUR®) from VWR Chemicals (Søborg, Denmark) and all were used without further purification. Dichloromethane (DCM, 99%) was obtained from Sigma-Aldrich and dried in a solvent distributor before use. Carbon monoxide (CO, 99.97%), hydrogen (H_2_, 99.999%), and nitrogen (N_2_, 99.99%) were obtained from Air Liquide Denmark (Taastrup, Denmark) and used as received. 

### 2.2. Cyclocarbonylation Reactions

In typical experiments, a solution of Pd(OAc)_2_ (0.02 mmol, catalyst/substrate molar ratio 4/1), DPEphos (0.08 mmol, ligand/substrate molar ratio 16/1), [BMIM]Cl (5.7 mmol), and 2-methyl-3-buten-2-ol (0.1 M, 0.5 mmol) in 5 mL of DCM was charged into customized stainless steel autoclaves (12 mL volume) equipped with manometers, thermo-elements, and pressure relief valves. After purging three times with N_2_ gas, the autoclaves were pressurized at room temperature with CO and H_2_ gases to a total pressure of 28 bar. Then the autoclaves were heated to a reaction temperature of 95 °C, where afterwards the reactions were continued for 18 h under stirring (700 rpm). When the reactions were completed, the autoclaves were cooled to room temperature in an ice bath, depressurized, and the reaction mixture analyzed by nuclear magnetic resonance (NMR) spectroscopy. 

For recycling experiments, the reaction mixture was extracted with DEE (3 × 5 mL) and both the combined extraction phases as well as the remaining catalyst-phase analyzed by NMR spectroscopy to confirm that all product was extracted successfully. Then, another amount of substrate was added to the catalyst-phase and reuse experiments performed by applying the same reaction procedure as described above. 

### 2.3. Product Analysis

The reaction products were dissolved in d-chloroform (CDCl_3_) and analyzed by high-resolution NMR spectroscopy at 400 MHz on a Bruker BioSpin GmbH NMR spectrometer (Solna, Sweden) operating at 25 °C using a 5 mm tunable multinuclear probe (PABBO BB-1H/D Z-GRD Z 116098/0150, Bruker, Solna, Sweden) with 25 s delay and 32 scans. Chemical shifts are referenced to CDCl_3_ at 7.26 ppm for proton NMR (^1^H NMR) and 77.16 ppm for carbon-13 NMR (^13^C NMR). The conversion of substrate and the yield of reaction were calculated using [BMIM]Cl (when present) as internal standard.

## 3. Results and Discussion

### 3.1. Effect of IL and Ligand Content

**Figure 1:**
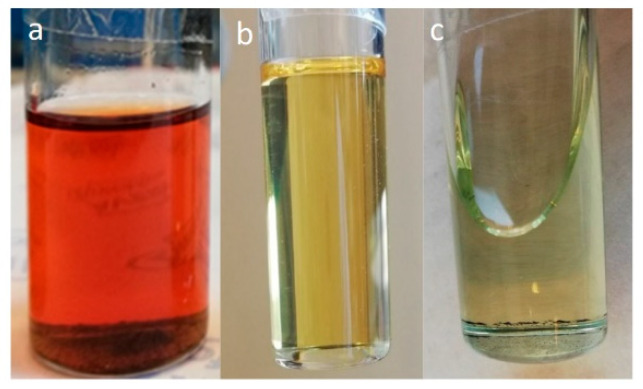
Reaction mixture after cyclocarbonylation of 2-methyl-3-buten-2-ol to 4,4-dimethyl-γ-butyrolactone (**a**) without IL and 16 mol% DPEphos, (**b**) with 1.0 g IL [BMIM]Cl and 16 mol% DPEphos, and (**c**) with 1.0 g IL [BMIM]Cl and 4 mol% DPEphos. Reaction conditions: 0.1 M 2-methyl-3-buten-2-ol, 4 mol% Pd(OAc)_2_, 5 mL DCM, 95 °C, 28 bar (CO/H_2_: 23/5), 18 h.

Initially, cyclocarbonylation with 2-methyl-3-buten-2-ol was performed in the absence of IL with 16 mol% DPEphos at a temperature of 95 °C with a total pressure of 28 bar (CO/H_2_: 23/5 bar) for 18 h, which was under much more benign conditions compared to previously reported for cyclocarbonylations with Pd-dppb [1,17]. Comparison of the ^1^H NMR spectra of the substrate and the crude reaction mixture (Figure S1) confirmed that the substrate was successfully converted to the desired 4,4-dimethyl-γ-butyrolactone product in high yield with only a low amount of byproduct formed. This byproduct could be 4-methyl-3-pentenoic acid formed by hydrolysis of the lactone or 3-methylbut-1-en formed by hydrogenation of the substrate. Although the Pd-catalyst system transformed the substrate well without the presence of IL, black precipitate was clearly visible in the solution after the reaction (Figure 1a). This showed that the Pd-DPEphos complexes were unstable under the reaction conditions and converted to inactive Pd-black (i.e. Pd metal), which could not be reused for consecutive cyclocarbonylation. When an analogous cyclocarbonylation reaction with 16 mol% DEPhos was performed with IL present (1 g of [BMIM]Cl), a high yield of the lactone product also formed as determined from NMR analysis of the reaction solution (Figure S2) and, importantly, the formation of Pd-black was successfully prevented leaving a clear yellow catalyst solution after the reaction (Figure 1b). In contrast, some Pd-black formation also occurred with a lower content of DEPhos corresponding to 4 mol% (Figure 1c), revealing that this ligand amount was insufficient to maintain the complexes active and stable despite the presence of IL [23]. Hence, the presence of [BMIM]Cl clearly stabilized the catalytically active system when sufficient ligand was available and thereby facilitated catalyst reuse after separation of the lactone product by, for example, extraction with DEE.

### 3.2. Effect of Reaction Temperature and Time

**Figure 2:**
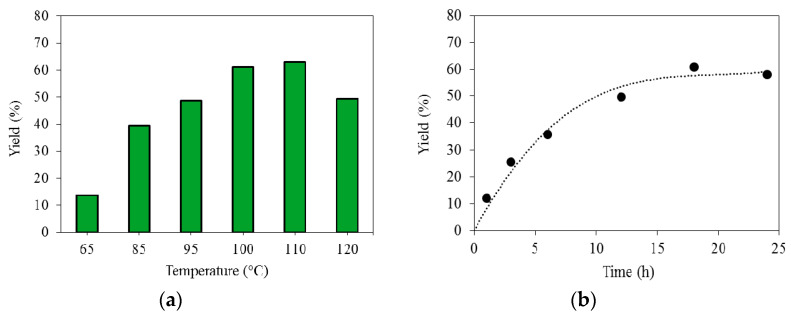
Effect of temperature (**a**) and reaction time (**b**) on the yield of 4,4-dimethyl-γ-butyrolactone formed by cyclocarbonylation of 2-methyl-3-buten-2-ol. Reaction conditions: 0.1 M 2-methyl-3-buten-2-ol, 4 mol% Pd(OAc)_2_, 16 mol% DPEphos, 1.0 g [BMIM]Cl, 5 mL DCM, 28 bar (CO/H_2_: 23/5), 18 h (**a**), 100 °C (**b**).

The effect of temperature on the yield of lactone product formed by the cyclocarbonylation reaction was examined at temperatures from 65 to 120 °C and the results are shown Figure 2a. A maximum lactone yield of about 65% was achieved at 100–110 °C, while the yield was lower at higher temperatures. This behavior was attributed to the formation of undesired byproducts at higher temperatures, such as 4-methyl-3-pentenoic acid. Accordingly, 100 °C was selected as the optimum temperature for all further studies. 

The lactone formation was next studied as a function of time at the preferred temperature of 100 °C. As shown in Figure 2b, the lactone formed relatively fast within the first 6 h where after the reaction became gradually slower reaching an almost constant plateau in yield around 60%–65% after 18 h of reaction. Based on this reaction profile, a reaction time of 18 h was selected as a good reaction time to study additional effects of the reaction system. 

### 3.3. Effect of Gas Composition

**Figure 3:**
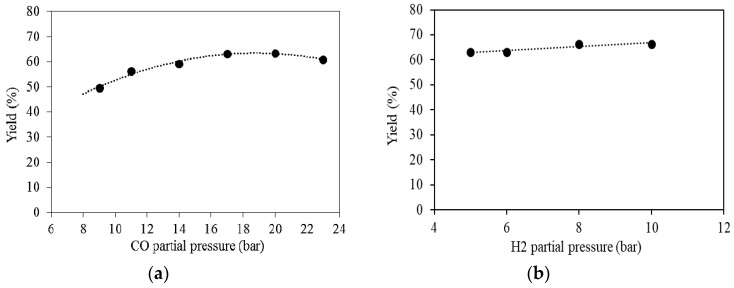
Effect of variable CO (**a**) and H_2_ (**b**) partial pressures on the yield of 4,4-dimethyl-γ-butyrolactone formed by cyclocarbonylation of 2-methyl-3-buten-2-ol. Reaction conditions: 0.1 M 2-methyl-3-buten-2-ol, 4 mol% Pd(OAc)_2_, 16 mol% DPEphos, 1.0 g [BMIM]Cl, 5 mL DCM, 100 °C, 28 bar ((**a**): 5 bar H_2_, (**b**): 18 bar CO), 18 h.

The influence of the CO/H_2_ gas composition on the yield of lactone product formed by the cyclocarbonylation is presented in Figure 3. To investigate the effect of the CO pressure on the reaction yield, its partial pressure was varied from 9 to 23 bar with a constant partial pressure of H_2_ of 5 bar (i.e., CO/H_2_ ratios 1.8–4.6) and the total pressure maintained at 28 bar with N_2_ as inert gas (Figure 3a). By increasing the partial pressure of CO from 9 to around 18–20 bar (corresponding to a CO/H_2_ ratio of 3.6–4), the yield of lactone increased while higher pressure of CO resulted in lower yields thus suggesting a complex reaction order with respect to the CO pressure. On the other hand, the effect of increasing the partial pressure of H_2_, relative to a fixed CO pressure, did not influence the lactone yield of the reaction therefore indicating a zero-order dependence with respect to the H_2_ pressure (Figure 3b).

**Scheme 2:**
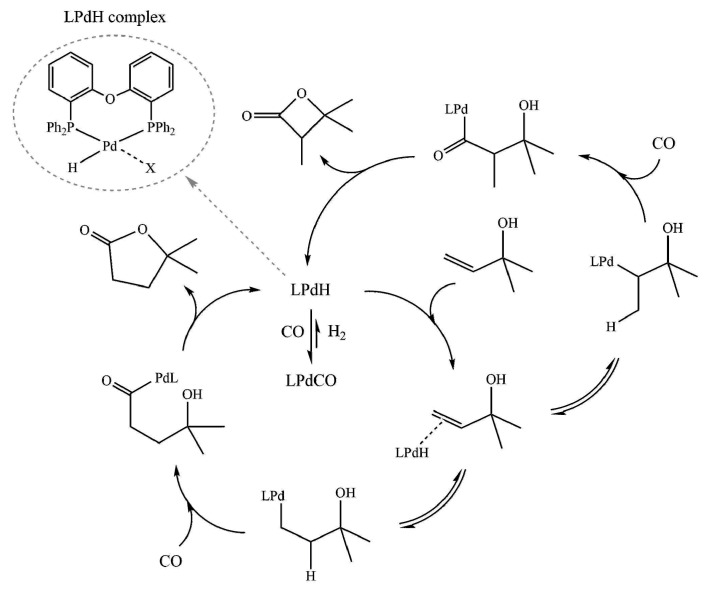
Suggested mechanism for the Pd-catalyzed cyclocarbonylation of 2-methyl-3-buten-2-ol forming 4,4-dimethyl-γ-butyrolactone with inserted structure of the L-Pd-H complex (Ph: phenyl, X: ancillary ligand).

The observed trends in pressure dependence corroborated well with a suggested reaction mechanism as shown in Scheme 2 [1,17], where H_2_ is only required to form active palladium hydride complex with ligand (L-Pd-H), but CO is involved in a carbonylation step as well as competitive conversion of the hydride complex into inactive palladium carbonyl complex with ligand (L-Pd-CO). In the proposed mechanism, the substrate initially added to the L-Pd-H complex followed by hydrogen migration, which can proceed through two different paths with formation of L-Pd-alkyl complex via α-addition to the double bond being most likely due to steric reasons. In the consecutive carbonylation step, CO is inserted forming a L-Pd-acyl complex which after ring closure and elimination of the lactone product regenerates the L-Pd-H complex.

### 3.4. Effect of Substrate Concentration 

The influence of substrate concentration on the substrate conversion and lactone yield were next studied using the optimal CO/H_2_ ratio of 4 at 28 bar (CO/H_2_/N_2_: 20/5/3 bar) and the results are compiled in Table 1. When the substrate concentration was increased from 0.43 to 0.52 mmol, the conversion as well as corresponding lactone yield decreased only gradually by 10% (70% to 60%) after 18 h of reaction, thus providing no indication of product inhibition of the catalyst system. Hence, prolongation of the reaction time at higher substrate conversion would most likely result in similar high conversion and yield as with lower substrate concentration. 

### 3.5. Reuse of the Catalytic System

As mentioned earlier, one of the most challenging issues in homogeneous catalytic systems used for cyclocarbonylation is the inability of recovering and re-using the precious catalyst for further reactions [17]. In the reaction concept studied in this work, the role of the Pd-DPEphos catalyst system (4 mol% Pd(OAc)_2_, 16 mol% DPEphos) containing the IL [BMIM]Cl was not only to make the reaction conditions milder, but also to stabilize and provide facile separation of the catalyst from the products. With this ability in mind, the reusability of the catalytic system was tested by using DEE (3 × 5 mL) as an extraction solvent. The recycling experiments were done under the same operating conditions used in the first run. Recycling of the IL catalytic system was studied by performing four consecutive reactions for 18 h with a CO/H_2_ ratio of 4 at 28 bar (CO/H_2_/N_2_: 20/5/3 bar) and a temperature of 100 °C. After each reaction the reactor was cooled down, depressurized, and lactone extracted (together with non-coordinated ligand) before new substrate was added and the reactor re-pressurized. 

**Figure 4:**
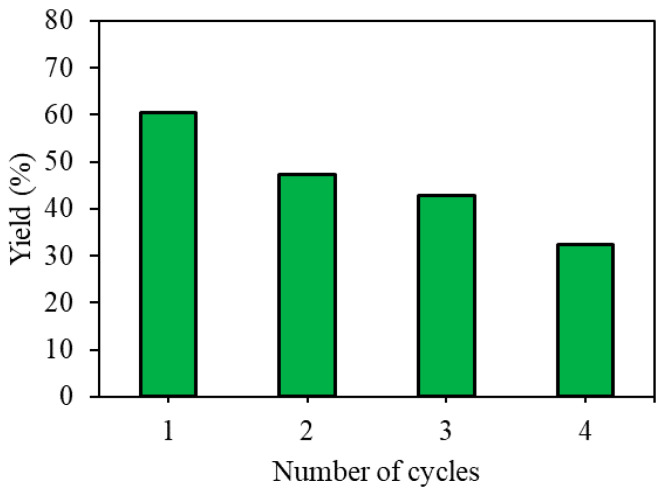
Recycling of the IL Pd-DPEphos catalyst system in the cyclocarbonylation of 2-methyl-3-buten-2-ol to 4,4-dimethyl-γ-butyrolactone. Reaction conditions: 0.1 M 2-methyl-3-buten-2-ol, 4 mol% Pd(OAc)_2_, 16 mol% DPEphos, 1.0 g [BMIM]Cl, 5 mL DCM, 100 °C, 28 bar (CO/H_2_/N_2_: 20/5/3), 18 h.

The results obtained from the reactions showed that the lactone yield decreased by an average of around 5% for each run from the first to the third run (from 60% to about 45%), whereas it decreased to around 35% after the fourth run as depicted in Figure 4. Importantly, Pd-black formation was only evident in the reaction solution after the fourth reaction run (Figure S3), suggesting that the Pd-complex remained quite stable in the first three runs as no visible precipitate formed after these runs. The formation of Pd-black has been investigated extensively in literature for homogeneous Pd-catalysts [22,32]. Garcia-Suarez et al. observed that the interaction of reactants, especially CO which is a well-known reducing agent with Pd(OAc)_2_, can lead to metal precipitation and thus lower the catalyst activity [22]. They attributed this phenomenon to *in situ* complex formation in the reaction mixture and in order to eliminate its effect, they suggested pre-formation of the catalytic mixture under the same conditions as the reaction but in the absence of reactants [22]. We also believe that the Pd-black formation after the third reaction was most likely due to reduction of the catalyst with the CO gas, when an insufficient amount of DPEphos ligand was left in the extracted catalyst phase to provide stabilization of the metal complex.

## 4. Conclusions

In this work, the cyclocarbonylation of unsaturated allylic alcohols, exemplified with 2-methyl-3-buten-2-ol to selectively produce 4,4-dimethyl-γ-butyrolactone, was studied under relatively mild reaction conditions using a Pd-DPEphos catalyst in the presence of the IL [BMIM]Cl. The presence of the IL successfully stabilized the catalyst system by preventing the formation of Pd-black when 16 mol% DPEphos ligand was present and facilitating product separation and catalyst recovery, while a lower amount of ligand of 4 mol% led to incomplete formation of catalytically active species resulting in decreased activity as well as stability. A reaction temperature of 100 °C was found to be optimal for obtaining near quantitative yield of 4,4-dimethyl-γ-butyrolactone, whereas formation of undesired byproducts (e.g., 4-methyl-3-pentenoic acid or 3-methylbut-1-en) increased at higher temperatures. Increase in H_2_ partial pressure higher than 5 bar did not have any significant effect on the yield of the cyclocarbonylation reaction, while the yield increased considerably when the partial pressure of CO was increased to 18–20 bar which corresponded to an optimal CO/H_2_ ratio around 4/1. At higher pressures of CO (i.e., higher CO/H_2_ ratios) lower yields of lactone were likely formed due to the formation of inactive Pd carbonyl complexes as also corroborated with a proposed reaction mechanism. Recycling experiments showed that the catalytic system with IL could be re-used for three reaction runs after product extraction without any formation of Pd-black, however a gradual decrease in activity occurred in the consecutive reaction runs due to simultaneous lowering of the amounts of DPEphos ligand. Hence, addition of a surplus of ligand after each reaction run could most likely circumvent catalyst deactivation upon recycling.

## Figures and Tables

**Table 1:** Effect of substrate amount on the cyclocarbonylation of 2-methyl-3-buten-2-ol ^1^.

Entry	2-methyl-3-buten-2-ol (mmol)	Conversion (%)	Yield of Lactone (%)	Selectivity (%)
1	0.43	98.1	69.7	71.0
2	0.46	96.8	63.5	65.6
3	0.48	91.4	60.0	65.6
4	0.52	88.8	58.1	65.4

^1^ Reaction conditions: 4 mol% Pd(OAc)_2_, 16 mol% DPEphos, 1.0 g [BMIM]Cl, 5 mL DCM, 100 °C, 28 bar (CO/H_2_/N_2_: 20/5/3), 18 h.

## Supplementary Materials

The following are available online at https://www.mdpi.com/1996-1944/13/9/2093/s1, Figure S1: ^1^H NMR spectra of (a) 2-methyl-3-buten-2-ol and (b) reaction mixture after cyclocarbonylation with the product 4,4-dimethyl-γ-butyrolactone, Figure S2: ^1^H NMR spectrum of the reaction mixture after cyclocarbonylation of 2-methyl-3-buten-2-ol with presence of the IL [BMIM]Cl and the product 4,4-dimethyl-γ-butyrolactone, Figure S3: Reaction mixture after (a) first reaction run (no Pd-black), (b) second reaction run (no Pd-black), (c) third reaction run (no Pd-black), and (d) fourth reaction run (Pd-black) during recycling of the IL Pd-DPEphos catalyst system in the cyclocarbonylation of 2-methyl-3-buten-2-ol.
