# Supplementary material for: Pd-Catalyzed Cyclocarbonylation of Allylic Alcohol under Benign Conditions with Ionic Liquid as Stabilizer
Source: Materials (Basel). 2020 May 1;13(9):2093. doi: 10.3390/ma13092093 (PMC7254326; doi:10.3390/ma13092093)
Supplement: Supplementary file 1 [file materials-13-02093-s001.pdf]

Article

Supporting Information

Pd-Catalyzed Cyclocarbonylation of Allylic Alcohol under Benign Conditions with Ionic Liquid as Stabilizer

Nasrin Nemati [1,2], Reza Eslamloueyan [1,*], Amalie Modvig [2] and Anders Riisager [2,*]

[1] Department of Chemical Engineering, School of Chemical and Petroleum Engineering, Shiraz University, Shiraz 71345, Iran; nasrinn@chalmers.se

[2] Centre for Catalysis and Sustainable Chemistry, Department of Chemistry, Technical University of Denmark, DK-2800 Kgs. Lyngby, Denmark; amaliemodvig@gmail.com

* Correspondence: eslamlo@shirazu.ac.ir (R.E.); ar@kemi.dtu.dk (A.R.); Tel.: +98-71-3613-3770 (R.E.); +45-4525-2233 (A.R.)

Received: 30 March 2020; Accepted: 28 April 2020; Published: date

(**a**)

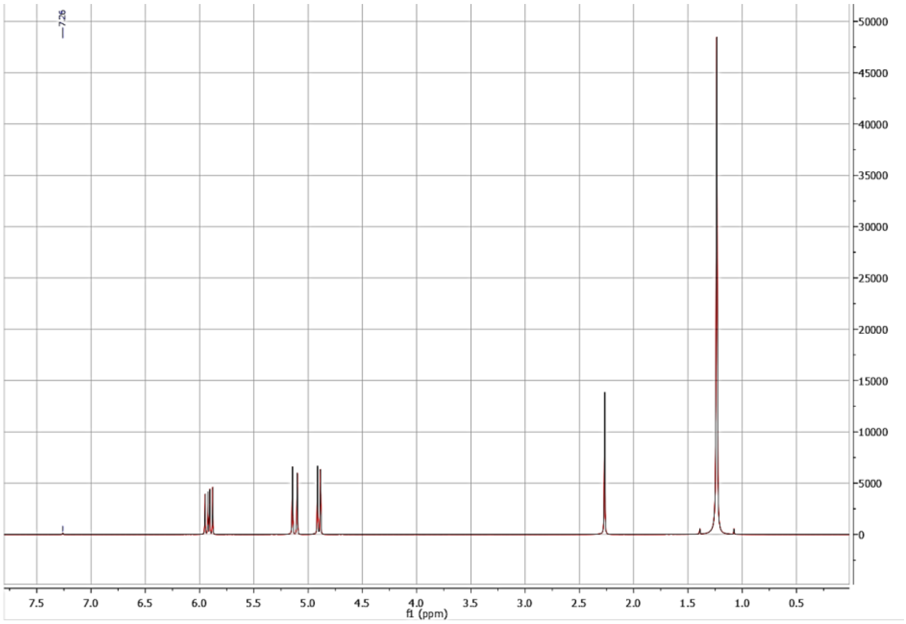

(**b**)

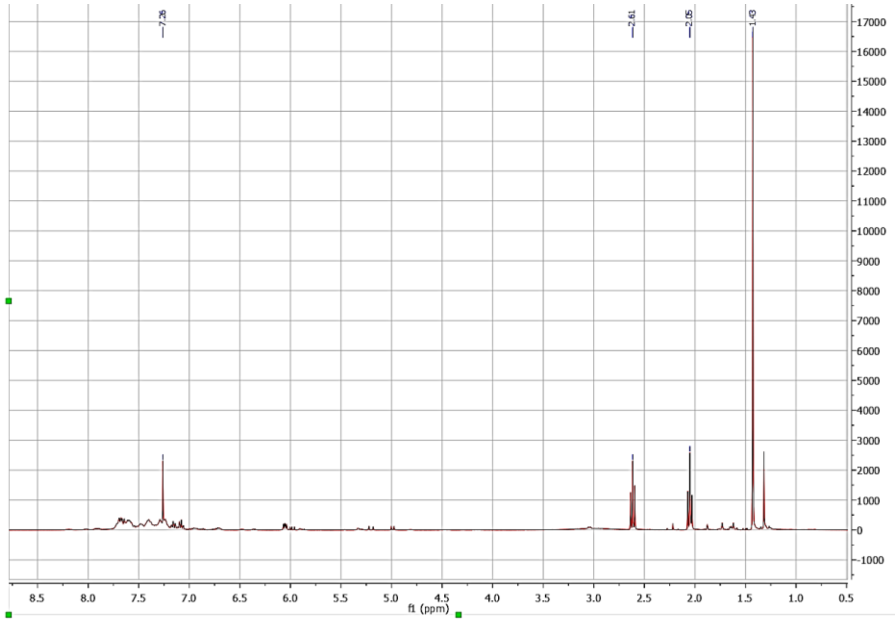

Figure S1. ^{1}H NMR spectra (400 MHz, $CDCl_3$) of (**a**) 2-methyl-3-buten-2-ol (δ/ppm: 1.2 (s, 2xCH_3, 6H), 2.3 (s, O*H*, 1H), 4.8 (d, *H*CH, 1H), 5.2 (d, HC*H*, 1H), 5.9 (dd, C*H*, 1H) and (**b**) reaction mixture after cyclocarbonylation with the product 4,4-dimethyl-γ-butyro-lactone (δ/ppm: 1.43 (s, CH_3, 6H), 2.05 (t, CH_2, 2H), 2.61 (t, COCH_2, 2H) and by-product(s) (δ/ppm: 1.63 (d), 3.06 (m), 5.28 (q)). Reaction conditions: 0.1 M 2-methyl-3-buten-2-ol, 4 mol% $Pd(OAc)_2$, 16 mol% DPEPhos, 5 ml DCM, 95 °C, 28 bar (CO/H_2: 23/5), 18 h.

materials

MDPI

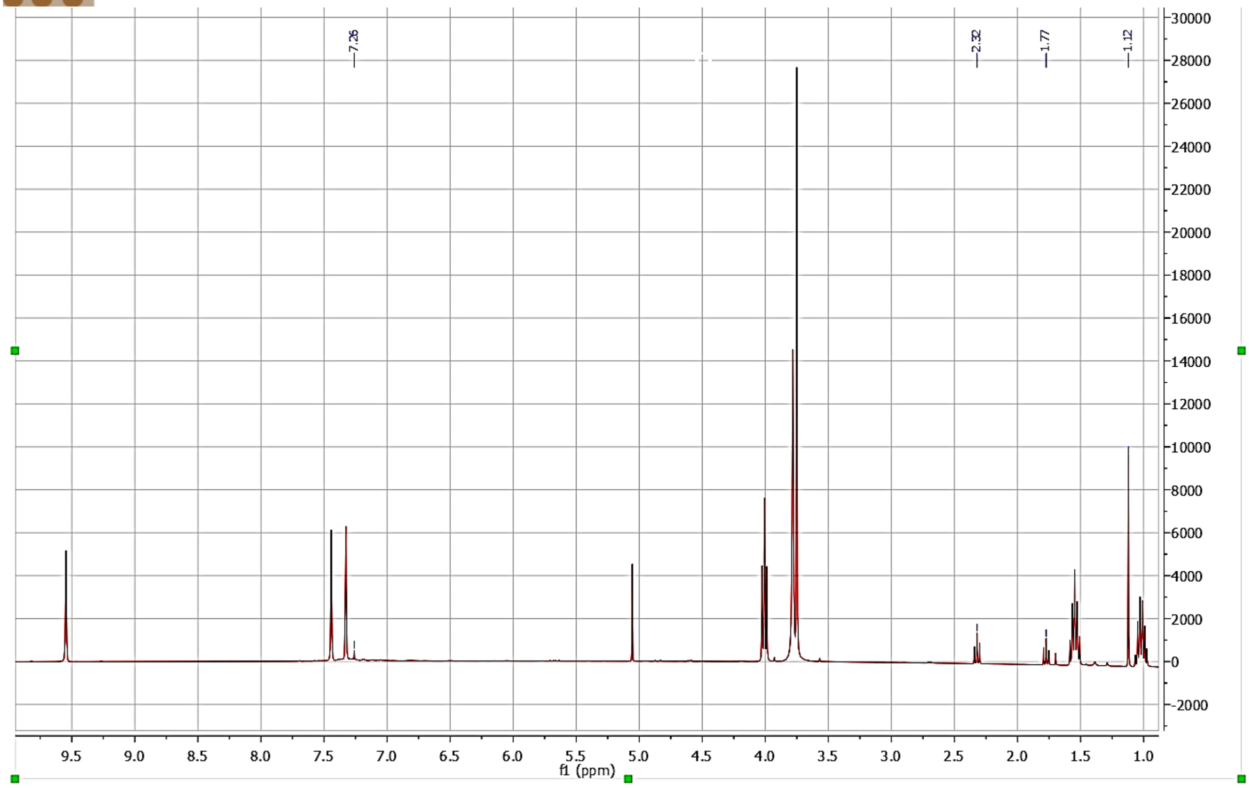

Figure S2. ^{1}H NMR spectrum (400 MHz, $CDCl_3$) of the reaction mixture after cyclocarbonylation of 2-methyl-3-buten-2-ol with presence of the IL [BMIM]Cl (δ/ppm: 1.05 (m, N-CH$_2$CH$_2$C*H*$_2$C*H*$_3$, 5H), 1.55 (q, N-CH$_2$C*H*$_2$CH$_2$CH$_3$, 2H), 4.0 (t, N-C*H*$_2$CH$_2$CH$_2$CH$_3$, 2H), 5.1 (s, N-C*H*$_3$, 3H), 7.3 (s, N-C*H*$_2$CH$_2$-N, 2H), 7.45 (s, N-CH$_2$C*H*$_2$-N, 2H), 9.6 (s, N-C*H*-N, 1H) and the product 4,4-dimethyl-γ-butyro-lactone (δ/ppm: 1.15 (s, C*H*$_3$, 6H), 1.75 (t, C*H*$_2$, 2H), 2.3 (t, COC*H*$_2$, 2H). Reaction conditions: 0.1 M 2-methyl-3-buten-2-ol, 1.0 g [BMIM]Cl, 4 mol% Pd(OAc)$_2$, 16 mol% DPEPhos, 5 ml DCM, 95 °C, 28 bar (CO/H$_2$: 23/5), 18 h.

(**a**)

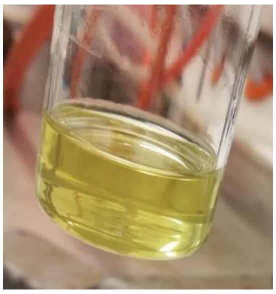

(**b**)

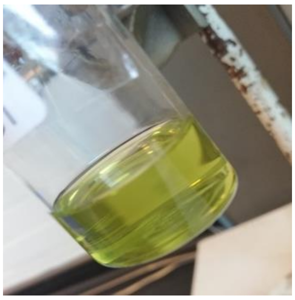

(**c**)

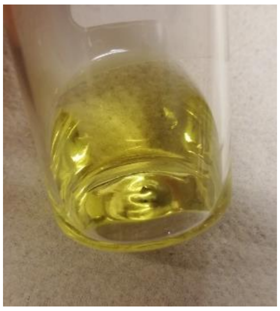

(**d**)

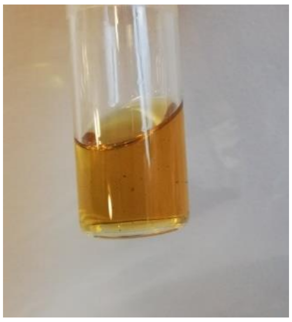

Figure S3. Reaction mixture after (**a**) first reaction run (no Pd-black), (**b**) second reaction run (no Pd-black), (**c**) third reaction run (no Pd-black) and (**d**) fourth reaction run (Pd-black) during recycling of the IL Pd-DPEPhos catalyst system in the cyclocarbonylation of 2-methyl-3-buten-2-ol. Reaction conditions: 0.1 M 2-methyl-3-buten-2-ol, 4 mol% $Pd(OAc)_2$, 16 mol% DPEPhos, 1.0 g [BMIM]Cl, 5 ml DCM, 100 °C, 28 bar ($CO/H_2/N_2$: 20/5/3), 18 h.

© 2020 by the authors. Submitted for possible open access publication under the terms and conditions of the Creative Commons Attribution (CC BY) license (http://creativecommons.org/licenses/by/4.0/).
